# Gender-Dependent Association of *FTO* Polymorphisms with Body Mass Index in Mexicans

**DOI:** 10.1371/journal.pone.0145984

**Published:** 2016-01-04

**Authors:** Yolanda Saldaña-Alvarez, María Guadalupe Salas-Martínez, Humberto García-Ortiz, Angélica Luckie-Duque, Gustavo García-Cárdenas, Hermenegildo Vicenteño-Ayala, Emilio J. Cordova, Marcelino Esparza-Aguilar, Cecilia Contreras-Cubas, Alessandra Carnevale, Margarita Chávez-Saldaña, Lorena Orozco

**Affiliations:** 1 Laboratorio de Immunogenómica y Enfermedades Metabólicas, Instituto Nacional de Medicina Genómica, Secretaría de Salud, Mexico City, Mexico; 2 Programa de Ciencias Genómicas, Universidad Autónoma de la Cd. de México, Mexico City, Mexico; 3 Hospital Regional 1° de Octubre, ISSSTE, Mexico City, Mexico; 4 Clínica de Diagnóstico Autorizado, ISSSTE, Mexico City, Mexico; 5 Hospital Regional Adolfo López Mateos, ISSSTE, Mexico City, Mexico; 6 Centro Nacional para la Salud de la Infancia y la Adolescencia, Secretaría de Salud, Mexico City, Mexico; 7 Instituto Nacional de Medicina Genómica, Secretaría de Salud, Mexico City, Mexico; 8 Instituto Nacional de Pediatría, Secretaría de Salud, Mexico City, Mexico; Johns Hopkins Bloomberg School of Public Health, UNITED STATES

## Abstract

To evaluate the associations between six single-nucleotide polymorphisms (SNPs) in intron 1 of *FTO* and body mass index (BMI), a case-control association study of 2314 unrelated Mexican-Mestizo adult subjects was performed. The association between each SNP and BMI was tested using logistic and linear regression adjusted for age, gender, and ancestry and assuming additive, recessive, and dominant effects of the minor allele. Association analysis after BMI stratification showed that all five *FTO* SNPs (rs1121980, rs17817449, rs3751812, rs9930506, and rs17817449), were significantly associated with obesity class II/III under an additive model (P<0.05). Interestingly, we also documented a genetic model-dependent influence of gender on the effect of *FTO* variants on increased BMI. Two SNPs were specifically associated in males under a dominant model, while the remainder were associated with females under additive and recessive models (P<0.05). The SNP rs9930506 showed the highest increased in obesity risk in females (odds ratio = 4.4). Linear regression using BMI as a continuous trait also revealed differential *FTO* SNP contributions. Homozygous individuals for the risk alleles of rs17817449, rs3751812, and rs9930506 were on average 2.18 kg/m^2^ heavier than homozygous for the wild-type alleles; rs1121980 and rs8044769 showed significant but less-strong effects on BMI (1.54 kg/m^2^ and 0.9 kg/m^2^, respectively). Remarkably, rs9930506 also exhibited positive interactions with age and BMI in a gender-dependent manner. Women carrying the minor allele of this variant have a significant increase in BMI by year (0.42 kg/m^2^, *P* = 1.17 x 10^−10^). Linear regression haplotype analysis under an additive model, confirmed that the *TGTGC* haplotype harboring all five minor alleles, increased the BMI of carriers by 2.36 kg/m^2^ (*P* = 1.15 x 10^−5^). Our data suggest that *FTO* SNPs make differential contributions to obesity risk and support the hypothesis that gender differences in the mechanisms involving these variants may contribute to disease development.

## Introduction

Obesity is a major risk factor for developing chronic degenerative disorders such as type 2 diabetes, hypertension, cardiovascular events, and some types of cancer[1, 2]. It is estimated that >50% of the global population will be overweight or obese by 2030 [3]. In Mexico, more than 70% of adults have a body mass index (BMI) ≥25 kg/m^2^, and 30% are obese (BMI ≥30 kg/m^2^) [4]. The growing prevalence of obesity and its co-morbidities worldwide in recent decades highlights the need to clarify the factors involved in its development. Genetic factors play a role in the etiopathogenesis of obesity, and many studies have shown that polymorphisms in candidate genes are associated with susceptibility to obesity. However, these observations have not been replicated in all populations, suggesting that ethnic differences may underlie the variability observed in association studies.

Recent genome-wide association studies (GWAS), have facilitated the identification of potential new genetic risk factors involved in the regulation of energy balance. The most-replicated finding is an association between fat mass and the obesity-associated gene *FTO*. Single-nucleotide polymorphisms (SNPs) clustered in the first intron of *FTO* display the strongest associations with obesity reported to date and have been investigated more than any other common variant in human obesity [5]. *FTO* encodes a demethylation enzyme that removes methyl groups from DNA and RNA nucleotides, and it is probably involved in physiological processes such as the control of energy homeostasis, adipogenesis, and DNA methylation. However, its role in the pathophysiology of obesity remains under investigation [5–8]. In murine models, *Fto* RNA transcript levels correlated with food intake, suggesting that this gene may participate in the central control of energy homeostasis [9, 10]. In addition, the FTO protein is expressed extensively in the mouse brain, where it serves as the main regulator of energy balance, evidencing a close association between FTO function and BMI regulation [11]. Furthermore, association studies indicated that subjects with at least one copy of the *FTO* risk allele of the SNP rs9939609, had higher food intake than those with two copies of the wild-type allele; the former also exhibited an increased preference for fatty food [12–14]. Case-control studies in individuals with insulin resistance, and other anthropometric measures such as weight and waist and hip circumference detected significant associations with polymorphisms on the first intron of *FTO* gene [15, 16]. The first study involving this *FTO* SNPs and obesity was performed in an English diabetic population; where the SNP most strongly associated with an increased BMI was rs9939609 [17]. Subsequently, other studies in German, French, and Italian populations reported that rs1121980 and rs9930506, were most strongly associated with obesity [18–20]. In Mexicans the rs9939609 was also associated with increased BMI [21, 22] but notably, rs1421085, which was associated with obesity in many populations, was not replicated in a cohort of obese Mexican children [23]. In order to increase our understanding of the contributions of this region of *FTO* to the development of obesity in the Mexican population, we performed a case-control study in a population that included normal-weight, overweight, and obese patients. We investigated the association of six SNPs distributed along intron 1 of *FTO gene*: rs7191566, rs1121980, rs1781449, rs3751812, rs9930506, and rs8044769, with obesity in a cohort of Mexican Mestizos (MMs).

## Materials and Methods

### Ethics statement

This study was conducted in accordance with the Declaration of Helsinki and was approved by the ethics and human research committees of the National Institute of Genomic Medicine in Mexico City, Mexico. All participants provided written informed consent.

### Study population

This study included 2314 unrelated MMs adults whose parents and grandparents were born in Mexico. Obesity status was determined according to World Health Organization criteria [24]. BMI was calculated as weight in kilograms divided by the square of height in meters. Based on BMI values, the subjects were categorized as normal weight (18.5–24.9 kg/m^2^), overweight (25–29.9 kg/m^2^), or obese (≥30 kg/m^2^). Blood pressure as well as fasting glucose, triglycerides, and cholesterol serum levels were measured in all participants. MM individuals were recruited from three tertiary health institutions in Mexico City: Hospital Regional 1° de Octubre, Hospital Regional Adolfo López Mateos, and Clínica de Diagnóstico Autorizado, ISSSTE. To determine the frequencies of the risk alleles in Mexican-Amerindians (MAs), we also included 592 MA adults who identified themselves as indigenous members of four ethnic groups (213 Mayas, 219 Nahuas, 83 Tarahumaras, and 77 Otomies). These participants were born in the same region as their parents and grandparents and both parents or all their grandparents speak a native language. Ancestry was confirmed in a random sample of 200 MAs and in the whole MMs sample, using 96 ancestry-informative markers (AIMs), by the Illumina GoldenGate microarray SNP genotyping method. These AIMs distinguish mainly between Amerindian and European ancestry (δ>0.44) and have been validated in other studies in Mexican population [25].

### Genotyping

Genomic DNA was extracted from whole blood cells using a high-salt method (QIAgen Systems, Inc., Valencia, CA, USA). Based on previous association data from several populations, we selected variants rs1121980, rs17817449, rs3751812, rs9930506, rs7191566, and rs8044769 [17–20] for analyses; these SNPs are distributed along a 45-kb stretch of intron 1 of *FTO*. Genotyping was performed with the TaqMan Allelic Discrimination assay using the ABI PRISM 7900 thermocycler (Applied Biosystems, Foster City, CA, USA). The call rate exceeded 96% for all SNPs tested, with no discordant genotypes in 15% of duplicate samples used as quality control. In order to validate the TaqMan results, random samples from each genotype were sequenced on an automated ABI PRISM 310 Genetic Analyzer (Applied Biosystems) with 100% reproducibility.

### Statistical analysis

Comparison of clinical data between cases and controls was carried out using the Kruskal-Wallis test. Associations between each *FTO* SNP and obesity were tested using logistic regression in PLINK v1.07 (http://pngu.mgh.harvard.edu/purcell/plink). All associations were evaluated under additive, dominant, and recessive inheritance models adjusted for *sex*, *age*, *and ancestry*. Odds ratios (ORs) with 95% confidence intervals and the Hardy—Weinberg equilibrium (HWE) were performed using FINETTI software. To investigate the impact of *FTO* polymorphisms on BMI, we used a linear regression model implemented in PLINK v1.07 with BMI as a continuous trait and age and sex as covariates [26]. Ancestry correction was performed via p*rincipal component analysis* in which eigenvectors were calculated from 96 AIMs with EIGENSOFT version 5.0. To assess the reliability of the differences observed among males and females, a test for heterogeneity was quantified by I^2^ measure [27]. Linkage disequilibrium (LD) structures and haplotype frequencies were analyzed using Haploview version 4.2 (http://www.broad.mit.edu/mpg/). Correction for multiple-hypothesis testing was performed with the Bonferroni correction and multiple permutation tests (100,000 permutations). Using QUANTO version 12 (http://hydra.usc.edu/GxE/), the statistical power for the study was estimated as >80% when employing an additive model. The level of statistical significance was defined as *P* values of ≤0.05 after Bonferroni correction.

## Results

### Study population

A total of 2314 MM unrelated adult subjects were included in this study. Of these, 1510 (65%) were women and 804 (35%) were men. Based on BMI, 698 (31%) were normal weight (18.5–24.9 kg/m^2^), 541 (23%) were overweight (25–29.9 kg/m^2^), and 1075 (46%) were obese *(≥30 kg/m*^*2*^). As expected, certain anthropometric and clinical values, such as blood pressure and serum levels of glucose, cholesterol, and triglycerides, were significantly higher (P<0.001) in obese and overweight subjects than in normal-weight individuals. Baseline characteristics of participants included in this study are shown in S1 Table.

### Association analysis in MMs

The genotype distribution of all evaluated SNPs was in Hardy-Weinberg equilibrium in our population, except for rs7191566, which was not considered in subsequent analysis. A comparative analysis between obese cases and normal-weight controls showed that the frequencies of all minor alleles were significantly associated with obesity after adjustments for gender, age, and ancestry rs1121980 (T): 0.27 vs. 0.24, OR = 1.2, *P* = 0.009; rs17817449 (G): 0.21 vs. 0.18, OR = 1.3, *P* = 0.007; rs3751812 (T): 0.20 vs. 0.16, OR = 1.4, *P* = 0.001; rs9930506 (G): 0.27 vs. 0.21, OR = 1.5, *P* = 0.001; and rs8044769 (C): 0.41 vs. 0.37, OR = 1.2, *P* = 0.01) (Table 1). For all SNPs, significant associations remained after Bonferroni correction. After BMI stratification, we observed that all studied SNPs were significantly associated with BMI >35 kg/m^2^ (class II/III), an effect that persisted after multiple-hypothesis correction (P≤0.05) (Table 1). The only SNP that was significantly associated with all obesity grades (class I-III) was rs9930506. Stratification by BMI and gender also revealed an influence of gender on the effect of these *FTO* variants on increased BMI in a genetic model-dependent manner (Table 2). After Bonferroni correction, rs1121980 and rs17817449 were significantly associated with class II/III obesity in women only when assuming an additive model (OR = 1.4, *P* ≤ 0.05), while rs3751812 and rs9930506 were associated with both women and men under different models (*P* ≤ 0.05) (Table 2). The strongest effect on obesity occurred with rs9930506 in women using a recessive model (OR = 4.4, 95% CI 1.40–14.10, *P* = 0.05). We found absence of heterogeneity between man and woman groups (I^2^ = 0), which support the fact that these SNPs have a stronger effect in women.

**Table 1:** Case-control association analysis of intron 1 *FTO* SNPs in MMs.

		MAF		Total Obesity			Class I Obesity			Class II/III Obesity		
SNP	Genetic model	Cases	Controls	OR (95% CI)	*P*	*P*_*corr*_	OR (95% CI)	*P*	*P*_*corr*_	OR (95% CI)	*P*	*P*_*corr*_
**rs1121980**	**Additive**	0.27	0.24	1.2(1.06–1.46)	0.009	0.045*	1.1(0.96–1.37)	0.81	NS	1.4(1.15–1.73)	0.001	0.005*
**C/T**	**Dominant**			1.3(1.06–1.57)	0.01	0.05*	1.2(0.94–1.47)	0.14	NS	1.5(1.16–1.93)	0.002	0.01*
	Recessive			1.4(0.90–2.04)	0.14	NS	1.18(0.74–1.87]	0.49	NS	1.6(0.99–2.70)	0.05	NS
**rs17817449**	**Additive**	0.21	0.18	1.3(1.07–1.50)	0.007	0.037*	1.2(0.96–1.43)	0.11	NS	1.4(1.16–1.81)	0.001	0.005*
**T/G**	**Dominant**			1.3(1.06–1.60)	0.01	0.05*	1.2(0.96–1.52)	0.10	NS	1.5(1.12–1.92)	0.005	0.025*
	Recessive			1.6(0.95–2.66)	0.07	NS	1.2(0.67–2.18)	0.52	NS	2.2(1.19–3.93)	0.01	0.05*
**rs3751812**	**Additive**	0.20	0.16	1.4(1.14–1.64)	0.001	0.005*	1.3(1.03–1.55)	0.03	NS	1.5(1.23–1.94)	0.0002	0.001*
**G/T**	**Dominant**			1.4(1.12–1.72)	0.002	0.01*	1.3(1.91–1.62)	0.04	NS	1.6(1.21–2.08)	0.0008	0.004*
	Recessive			1.9(1.05–3.30)	0.03	NS	1.6(0.82–2.96)	0.17	NS	2.3(1.17–4.43)	0.02	NS
***rs9930506***	***Additive***	0.27	0.21	1.5(1.17–1.84)	0.001	0.005*	***1*.*4(1*.*07–1*.*79)***	***0*.*01***	*0*.05*	***1*.*6(1*.*23–2*.*13)***	***0*.*001***	*0*.005*
***A/G***	**Dominant**			1.5 (1.16–1.99)	0.002	0.01*	*1*.*2(1*.*04–1*.*94)*	0.03	NS	*1*.*7(1*.*21–2*.*36)*	0.002	0.01*
	Recessive			2.0(1.07–3.62)	0.03	NS	1.7(0.89–3.50)	0.10	NS	*2*.*3(1*.*13–4*.*70)*	0.02	NS
**rs8044769**	**Additive**	0.41	0.37	1.2(1.04–1.36)	0.01	0.05*	1.1(0.98–1.33)	0.09	NS	1.3(1.06–1.51)	0.01	0.05*
**T/C**	Dominant			1.3 (1.02–1.53])	0.03	NS	1.19(0.95–1.48)	0.13	NS	1.4(1.05–1.8)	0.02	NS
	Recessive			1.3 (0.98–1.66)	0.06	NS	1.2(0.89–1.60)	0.22	NS	1.4(0.97–1.90)	0.07	NS

* and bold font denotes significant *P* values (<0.05) after Bonferroni correction (*P*_corr_).

Italics and bold font denote significant associations with all classes of obesity. By convention, in all models, 1 is the major allele and 2 is the minor allele. Additive model; 11 versus 22; dominant model; 11+12 versus 22; recessive model; 11 versus 12+22. CI, confidence interval;MMs Mexican Mestizo. NS, not significant. MAF, minor allele frequency.

**Table 2:** Association of intron 1 *FTO* SNPs with obesity grade, stratified by gender, in MMs.

		Class I Obesity						Class II/III Obesity					
	Genetic	Female			Male			Female			Male		
SNP	model	OR (95% CI)	*P value*	*P*_*corr*_	OR (95% CI)	*P value*	*P*_*corr*_	OR (95% CI)	*P value*	*P*_*corr*_	OR (95% CI)	*P value*	*P*_*corr*_
**rs1121980**	**Additive**	1.1(0.89–1.42)	0.32	NS	1.1(0.86–1.51)	0.36	NS	1.4(1.10–1.79)	0.01	0.05*	1.4(0.92–2.01)	0.12	NS
**C/T**	Dominant	1.1(0.85–1.49)	0.39	NS	1.2(0.86–1.73)	0.27	NS	1.4(1.06–1.92)	0.02	NS	1.6(0.96–2.64)	0.07	NS
	Recessive	2.3(0.68–2.39)	0.44	NS	1.0(0.52–2.07)	0.91	NS	1.9(1.05–3.57)	0.03	NS	1.1(0.46–2.88)	0.76	NS
**rs17817449**	**Additive**	1.1(0.87–1.46)	0.35	NS	1.2(0.90–1.69)	0.52	NS	1.4(1.07–1.79)	0.01	0.05*	1.6(1.05–2.46)	0.03	NS
**T/G**	Dominant	1.2(0.87–1.58)	0.29	NS	1.2(0.86–1.80	0.94	NS	1.4(1.01–1.88)	0.04	NS	1.7(1.02–2.88)	0.04	NS
	Recessive	1.0(0.40–2.26)	0.94	NS	1.5(0.61–3.73)	0.37	NS	2.2(1.08–4.53)	0.03	NS	2.1(0.72–6.21)	0.17	NS
**rs3751812**	**Additive**	1.2(0.92–1.56)	0.18	NS	1.4(0.98–1.88)	0.06	NS	1.4(1.07–1.85)	0.01	0.05*	1.9(1.24–2.96)	0.003	0.015*
**G/T**	***Dominant***	1.2(0.90–1.65)	0.19	NS	1.4(0.93–2.00)	0.11	NS	1.4(1.02–1.92)	0.04	NS	2.2(1.30–3.67)	0.003	0.015*
	Recessive	1.3(0.57–3.11)	0.49	NS	1.9(0.71–5.19)	0.19	NS	2.3(1.03–5.15)	0.04	NS	2.1(0.65–7.01)	0.21	NS
**rs9930506**	Additive	1.4(0.94–2.00)	0.09	NS	1.4(0.96–1.95)	0.09	NS	1.6(1.09–2.23)	0.01	0.05*	1.7(1.08–2.60)	0.02	NS
**A/G**	***Dominant***	1.3(0.84–2.02)	0.22	NS	1.5(0.97–2.38)	0.07	NS	1.5(0.95–2.20)	0.08	NS	2.2(1,22–3.82)	0.008	*0*.04*
	**Recessive**	2.9(0.88–9.93)	0.08	NS	1.3(0.56–3.11)	0.52	NS	4.4 (1.4–14.1)	0.01	0.05*	1.3(0.45–3.60)	0.64	NS
**rs8044769**	Additive	1.2(0.95–1.42)	0.14	NS	1.1(0.85–1.38)	0.51	NS	1.3(1.03–1.57)	0.03	NS	1.2(0.85–1.70)	0.30	NS
**T/C**	Dominant	1.2(0.94–1.65)	0.13	NS	1.0(0.74–1.54)	0.71	NS	1.3(0.96–1.79)	0.08	NS	1.5(0.84–2.53)	0.17	NS
	Recessive	1.1(0.80–1.75)	0.40	NS	1.1(0.76–1.84)	0.44	NS	1.5(0.99–2.23)	0.05	NS	1.0(0.58–2.02)	0.79	NS

* and bold font denotes significant *P* values (<0.05) after Bonferroni correction (*P*_corr_).

By convention, in all models, 1 is the major allele and 2 is the minor allele. Additive model, 11 versus 22; dominant model, 11+12 versus 22; recessive model, 11 versus 12+22. CI, confidence interval; MMs Mexican Mestizo. NS, not significant.

#### Linear regression analysis

Linear regression using BMI as a continuous trait (including overweight subjects) and an additive model adjusted for sex, age, and ancestry, revealed differential SNP contributions to increased BMI. MMs homozygous for the risk alleles of rs17817449, rs3751812, and rs9930506 were on average *2*.*18* kg/m^2^ heavier than MMs homozygous for the wild-type alleles. In contrast, rs1121980 and rs8044769, located toward the 5’ and 3’ ends of *FTO*, respectively, had less influence on BMI (1.54 kg/m^2^ and 0.9 kg/m^2^, respectively) than variants located between them (Table 3). After stratification for age and gender, linear regression showed that rs1121980 was significantly associated with obesity in females only (beta = 0.89, *P* = 0.01), while the other SNPs displayed association in both genders. We also documented a positive interaction between age and BMI in women carrying the minor allele of rs9930506G (*P* = 5.4 x 10^−9^). Linear regression haplotype analysis confirmed that haplotype TGTGC, which harbors all five minor alleles, increased the BMI of carriers by 2.36 kg/m^2^ (*P* = 1.15 x 10^−5^) (Table 4*)*. Linear regression did not uncover any significant associations between first-intron *FTO* variants and blood pressure or serum levels of glucose, cholesterol, and triglycerides.

**Table 3:** Linear regression analysis of *FTO* SNPs in MMs, adjusted for gender, age, and ancestry.

SNP ID	N	Location							
Alleles	2314	Chr:16	MAF	TEST	BETA	L95	U95	*P*_*value*_	*P*_*corr*_
rs1121980 C/T	All	52366748	T	ADD	0.77	0.342	1.198	0.0004	0.002*
rs17817449 T/G	All	52370868	G	ADD	1.00	0.527	1.476	3.6 x 10^−5^	0.0002*
rs3751812 G/T	All	52375961	T	ADD	1.11	0.621	1.597	9 x 10^−6^	**4.5 x 10**^−5^*
rs9930506 A/G	All	52387966	G	ADD	1.17	0.426	1.910	0.002	*0*.01*
rs8044769 T/C	All	52396636	C	ADD	0.450	0.084	0.825	0.02	0.10

* and Bold font denotes significant P values (<0.05) after Bonferroni correction (P_corr_).

ADD, additive model. MMs Mexican Mestizo. MAF, minor allele frequency.

**Table 4:** *FTO* haplotype association with obesity in MMs.

	*Logistic regression analysis*^a^				*Lineal regression analysis*^b^			
Haplotype	Frequencies	OR	*P*_*value*_	*P*_*corr*_	Frequencies	BETA	*P*_*value*_	*P*_*corr*_
**TGTGC**	0.17	1.4	0.0004	0.002*	0.17	1.18	2.34X10^6^	**1.15X10**^5^*
TTGGC	0.05	1.01	0.95	NS	0.05	-0.488	0.27	NS
TGGAC	0.01	0.51	0.02	NS	0.01	-1.19	0.16	NS
CTGAC	0.15	1.04	0.71	NS	0.15	-0.167	0.54	NS
**CTGAT**	0.59	0.84	0.01	0.05*	0.59	-0.51	0.008	0.04*

Bold font denotes haplotypes with significant

*P values <0.05 after Bonferroni correction.

^a^Analysis including cases and controls.

^b^Analysis including obese, overweight and controls subjects.

MMs Mexican Mestizo. NS, not significant.

#### Allele frequencies

Comparisons between-population indicated that the frequencies of minor alleles in normal-weight MMs (rs1121980 (T), 0.24; rs17817449 (G), 0.18; rs3751812 (T), 0.16; and rs9930506 (G), 0.21), were significantly lower than those previously reported for Caucasians (0.48, 0.46, 0.46, and 0.48, respectively; *P* < 0.001), but similar to those previously reported for Japanese subjects (0.22, 0.18, 0.18, and 0.23, respectively) and Chinese subjects (0.21, 0.17, 0.18, and 0.23, respectively). The frequency of the rs8044769 risk allele was significantly lower (*P* < 0.001) in MMs (0.37) and in individuals from Los Angeles of Mexican origin (0.46) than in Caucasian, Japanese, and Chinese individuals, according to HapMap (Table 5). In order to know the Amerindian influence on the BMI in Mexicans, we analyzed a sample of MAs to investigate the allele frequencies of the three SNPs that more strongly affected BMI in MMs. Notably, all risk alleles displayed the lowest frequencies in MAs (rs1781449 (G), 0.07; rs3751812 (T), 0.06; and rs9930506 (G), 0.11, versus frequencies reported for other populations, including that observed here for MMs, which was intermediate between those of Caucasians and MAs (Table 5).

**Table 5:** Comparison of risk allele frequencies of intron 1 *FTO* SNPs among different ethnicities.

		Risk Allele Frequencies										
SNP	Risk allele	YRB	CHB	JPT	CEU	MXL	MMs	MAs	^a^*P*	^b^*P*	^c^*P*	^d^*P*
rs1121980	***T***	0.47	0.21	0.22	0.48	0.24	0.24	NA		< 0.001	< 0.001	> 0.05
rs17817449	***G***	0.39	0.17	0.18	0.46	0.18	0.18	0.07	< 0.001	< 0.001	< 0.001	> 0.05
rs3751812	***T***	0.07	0.18	0.18	0.46	0.17	0.16	0.06	< 0.001	< 0.001	<0.003	> 0.05
rs9930506	***G***	0.19	0.24	0.23	0.48	0.22	0.21	0.11	< 0.001	< 0.001	0.72	> 0.05
rs8044769	***C***	0.83	0.57	0.68	0.56	0.46	0.37	NA		< 0.001	< 0.001	< 0.001

^a^MMs versus MAs.

^b^MMs versus Caucasians.

^c^MMs versus Yorubas.

^d^MMs versus Chinese and Japanese populations.

NS, not significant. NA, not analyzed. MMs Mexican Mestizo. MAs, Mexican Amerindian.

**Fig 1:**
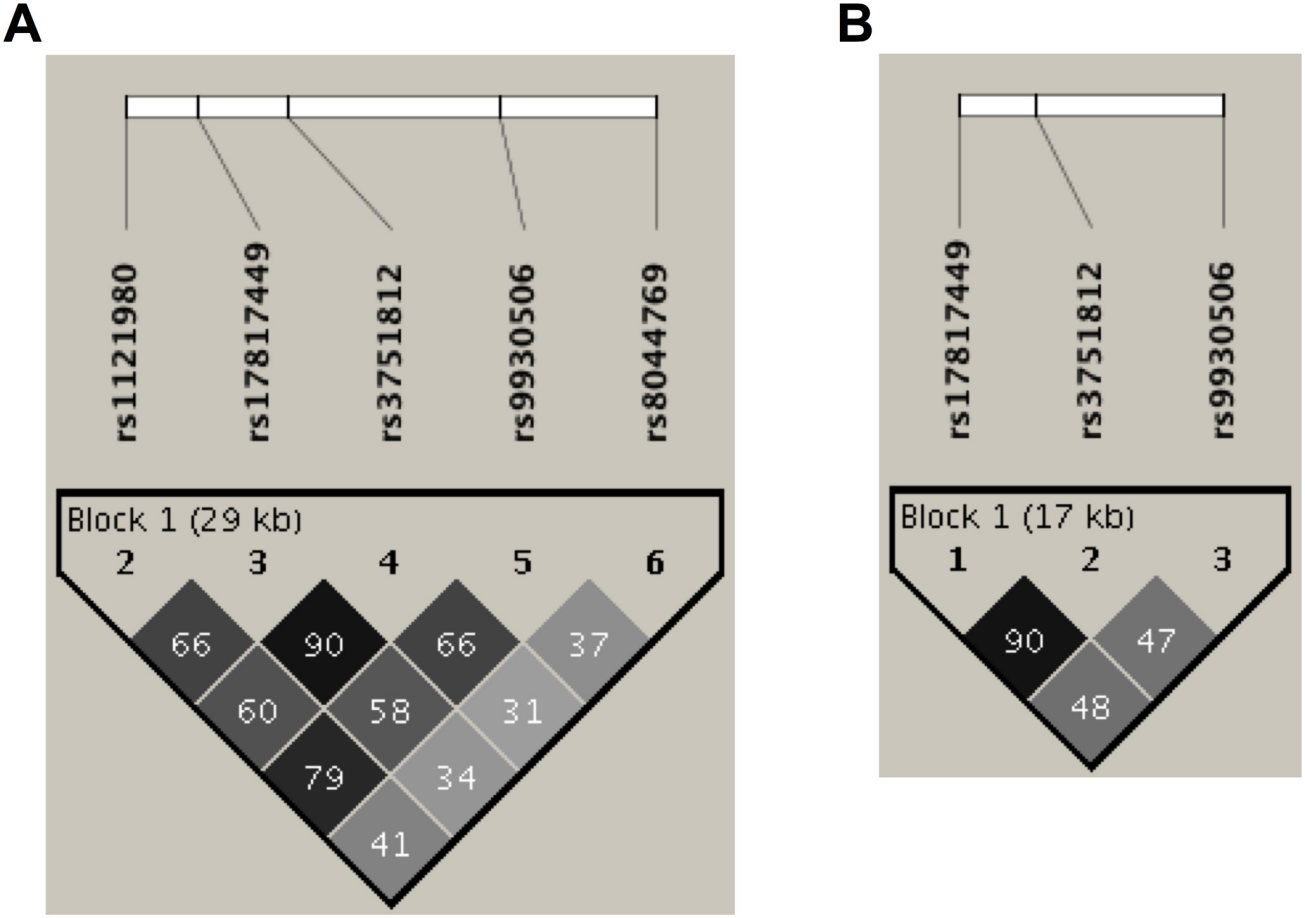
LD structure of SNPs in intron 1 of *FTO* in A) MMs and B) MAs. Black represents very high LD (r^2^) and white indicates a lack of correlation between SNPs. r^2^ was calculated with Haploview 4.2.

Interestingly, MMs and MAs showed different LD blocks. In the MM population included in this study, all SNPs were in LD (r^2^ = 0.90–0.66) except for rs8044769 (r^2^ = 37), which displayed a rapid fall-off downstream of the middle region (Fig 1). In contrast, in the MAs studied here, only two of three variants were in LD (rs17817449 and rs3751812: r^2^ = 90) (Fig 1).

## Discussion

The association of *FTO* with obesity is strongly replicable in most populations. However, the molecular mechanisms by which *FTO* variants increase individual susceptibility to overweight and obesity remain unclear. We performed a study in patients with a BMI ≥25 in which we analyzed six SNPs (rs7191566, rs1121980, rs17817449, rs3751812, rs9930506, and rs8044769) distributed across a 45-kb stretch of intron 1 of *FTO*. The rs7191566 was excluded from our analysis because it displayed no-HWE in women controls. In our MM population, we detected strong associations between all five *FTO* SNPs and *obesity* [17–19, 24]. Interestingly, after BMI stratification, it was notable that SNPs located in a 9-kb region in the middle of intron 1 (rs1121980, rs17817449, and rs3751812) with high LD (r^2^ = 90) were associated only with class II/III obesity. In contrast, rs9930506 was significantly associated with all obesity grades (class I-III) and was the only SNP showing a positive interaction between age and BMI, like the described in Italian individuals [28]. Otherwise, rs8044769 located near the 3’ end of intron 1 and which is in lower LD than the other SNPs (r^2^ = 37), showed only a nominal association with obesity after Bonferroni correction.

Notably, linear regression revealed that variants located in the middle of intron 1 (rs17817449, rs3751812, and rs9930506) more strongly influenced BMI than did SNPs toward the 5’ (rs1121980: beta = 0.77) and 3’ (rs17817449: beta = 0.45) ends.

In accordance with our observations, Bell et al. [29] recently reported that the CGCTTGG haplotype, which contains the minor alleles rs1421085 (T/C), rs17817449 (T/G), rs8050136 (A/C), rs3751812 (G/T), rs9939609 (A/T), rs7202116 (*A/G)*, *and* rs9930506 (A/G), harbors a specific methylation region. Actually, risk allele rs9930506G could has the potential to create or abrogate a CpG site in *FTO*, as has been described for the rs7202116G *FTO* allele [29]. Therefore, we cannot rule out the possibility that the risk haplotype *TGTGC* identified in the present study may influence the *FTO* methylation pattern and thus *FTO* expression.

*FTO* is expressed mainly in the hypothalamus and may be crucial for energetic homeostasis and lipolysis. Moreover, Almén et al. [30] recently provided evidence that *FTO* influences the methylation of genes that encode transcriptional regulators, such as *KARS* and *TERF2IP*, as well as the methylation of transcriptional coactivators that are induced by estrogen, such as *BCAS3*. Thus, it is possible that interactions between genetic and epigenetic factors may determine genetic susceptibility to obesity through *FTO*. Furthermore, based on the rapid fall-off of LD downstream of the region that contains the SNPs that consistently display associations with obesity, it is highly likely that functional variants are located within this *FTO* region. Another interesting finding in the present study was the genetic model-dependent sexual dimorphism for obesity susceptibility due to *FTO*. *FTO* SNPs were associated with obesity in females under both additive and recessive models and with males under a dominant model. These sex-based differences in the heritability of an increased BMI are consistent with previous studies that reported that *FTO* association with obesity traits occurs independently in males and females [31–33]. Moreover, a meta-analysis of 32 GWAS of waist-hip ratio adjusted for BMI identified seven loci that showed marked sexual dimorphism; all loci exerted stronger effects on waist-hip ratios in women than in men [34]. Taken together, these findings provide strong evidence that gene-by-sex interactions are involved in the distribution of body fat [34]. Since the majority of women who participated in our study are undergoing hormonal decline (mean age, 45.4±9.6 years), we cannot rule out the possibility that the association observed in this study is influenced by estrogen deficiency, as reported previously [35–37]. In fact, several studies have demonstrated that estrogen deficiency is responsible for the progression of sexually dimorphic obesity. Nevertheless, a relationship between *FTO* and the expression of sexual hormones has also been reported. For example, Zhang et al. [38] reported that estradiol (E2) induced *FTO* expression via the PI3K/AKT and MAPK signaling pathways; uncovering also a positive relationship among estrogen, *FTO* mRNA levels, and obesity. Hence, it is possible that genes regulated by sex-specific factors may underlie the sex-based differences in susceptibility to obesity observed in the present investigation. Further research is needed to confirm this hypothesis and to define the mechanism(s) involved. Overall, and consistent with most previous studies, our results strongly indicate that *FTO* has a very close relationship with obesity and fat mass, more than with other metabolic traits. The frequencies of the minor alleles of the five *FTO* SNPs analyzed in our MM sample were significantly lower than those reported for Caucasian populations. We included a MA sample to investigate the frequencies of the three SNPs (rs17817449 (G), rs3751812 (T), and rs9930506 (G) with the highest effect on obesity in MMs. Interestingly, in our indigenous population, all minor alleles of these three SNPs were present at the lowest frequencies reported worldwide. A recent investigation documented ethnicity-dependent differences in genetic susceptibility to obesity across populations, they reported evidence of a higher impact of *FTO* on Caucasians than on Chinese, African, and Hispanic populations [33]. Given these data and the differences in the LD structure of *FTO* SNPs between MMs and MAs observed here, it is necessary to investigate whether *FTO* influences obesity risk in the indigenous population of Mexico. Our results suggest that the contribution of *FTO* to increased BMI in MMs stems more strongly from their Caucasian heritage than from their Amerindian heritage.

In conclusion, our results demonstrate that SNPs in *FTO* exerts differential impacts on BMI. Also, our data strongly support the hypothesis that sex plays an important role in the relationship between *FTO* SNPs and the development of obesity.

Identifying and understanding the mechanisms that underlie the relationship between *FTO* and obesity will facilitate the development of rational strategies for personalized management of this metabolic disease.

## Supporting Information

S1 Table (DOC)
