# Supplementary material for: Gender-Dependent Association of FTO Polymorphisms with Body Mass Index in Mexicans
Source: PLoS One. 2016 Jan 4;11(1):e0145984. doi: 10.1371/journal.pone.0145984 (PMC4703196; doi:10.1371/journal.pone.0145984)
Supplement: S1 Table — (DOC) [file pone.0145984.s001.doc]

**Table S1. Clinical characteristics of the Mexican-Mestizos included in this study.**

| **Mexican-Mestizo N = 2314** | | | | |
| --- | --- | --- | --- | --- |
|  | **Normal weight** | **Over weight** | **Obese** | **P-value** |
| Women, n (%) | 432(62%) | 353(65%) | 725(67%) | 0.13 |
| Men, n (%) | 266(38%) | 188(35%) | 350(33%) | 0.13 |
| **BMI in kg/m^2^** |  |  |  |  |
| Women, mean±SD | 23.1(±1.6) | 27.7(±1.1) | 37.0(±5.9) | <0.0001 |
| Men, mean±SD | 23.5(±1.4) | 27.8(±1.1) | 34.3(±4.7) | <0.0001 |
| Age in years, mean±SD | 47.9(±12.9) | 54.42(±9.9) | 48.1(±10.6) | <0.0001 |
| Fasting glycemia in mg/dL, mean±SD | 113.1(±12.1) | 126.4(±60.0) | 122.1(±54.1) | <0.0001 |
| Cholesterol in mg/dL, mean±SD | 168.5.1(±72.5) | 203.5(±47.6) | 198.8(±69.9) | <0.0001 |
| Triglycerides in mg/dL, mean±SD | 128.9(±111.6) | 152.8(±116.8) | 130.1(±89.2) | <0.0001 |
| Systolic BP in mmHg, mean±SD | 117.7 (±11.7) | 128.9.0(±22.7) | 127.2(±17.9) | <0.0001 |
| Diastolic BP in mmHg, mean±SD | 73.8 (±9.4) | 76.9(±12.8) | 80.7(±10.4) | <0.0001 |

**P values were analyzed using the chi-squared test for trend for categorical data and the Kruskal-Wallis test. BP, blood pressure; SD, standard deviation.**
